# Essential Oils from the Leaves, Stem, and Roots of *Blumea lanceolaria (Roxb.)* Druce in Vietnam: Determination of Chemical Composition, and In Vitro, In Vivo, and In Silico Studies on Anti-Inflammatory Activity

**DOI:** 10.3390/molecules27227839

**Published:** 2022-11-14

**Authors:** Thi Thanh Huyen Do, Thi Uyen Nguyen, Thi Thu Huyen Nguyen, Thi Yen Ho, Thi Luong Hang Pham, Tho Son Le, Thi Hong Van Nguyen, Phi-Hung Nguyen, Quang Huy Nguyen, Van Sang Nguyen

**Affiliations:** 1 Faculty of Biology, University of Science, Vietnam National University, Hanoi, 334 Nguyen Trai, Thanh Xuan, Hanoi 100000, Vietnam; 2 HUS High School for Gifted Students, University of Science, Vietnam National University, 182 Luong the Vinh, Thanh Xuan, Hanoi 100000, Vietnam; 3 Molecular and Cellular Biology Laboratory, Center for Life Science, Faculty of Biology, University of Science, Vietnam National University, 334 Nguyen Trai, Thanh Xuan, Hanoi 100000, Vietnam; 4 Department of Molecular Genetics and Gene Technology, College of Forestry Biotechnology, Vietnam National University of Forestry, Xuan Mai, Chuong My, Hanoi 100000, Vietnam; 5 Institute of Natural Products Chemistry, Vietnam Academy of Science and Technology (VAST), 18 Hoang Quoc Viet, Cau Giay, Hanoi 122100, Vietnam; 6 Department of Chemistry, Graduate University of Science and Technology, Vietnam Academy of Science and Technology (VAST), 18 Hoang Quoc Viet, Cau Giay, Hanoi 122100, Vietnam; 7 National Key Laboratory of Enzyme and Protein Technology, University of Science, Vietnam National University, 334 Nguyen Trai, Thanh Xuan, Hanoi 100000, Vietnam

**Keywords:** *Blumea lanceolaria (Roxb.)*, essential oils, anti-inflammation, NF-κB, p38MAPK, COX-2, molecular docking

## Abstract

*Blumea lanceolaria (Roxb.)* Druce, a flowering plant, is used for treating cancer and inflammatory diseases. In this study, we determined the chemical composition of the EOs extracted from the leaves (LBEO), stem (SBEO), and roots (RBEO) of *B. lanceolaria* and analyzed their anti-inflammation potential. Overall, 30 compounds representing 99.12%, 98.44%, and 96.89% of total EO constituents of the leaves, stem, and roots, respectively, were identified using GC-MS. ELISA, Western blotting, and qRT-PCR studies showed that LBEO, SBEO, and RBEO inhibited multiple steps in the inflammatory responses in the RAW 264.7 cell model, including NO production; TNF-α, IL-6, iNOS, and COX-2 transcription and translation; and phosphorylation of IκBα and p65 of the NF-κB pathway. In the carrageenan-induced paw edema model, all three EOs inhibited paw edema at both early and delayed phases. Molecular docking studies indicated that the main components of *B. lanceolaria* EOs (BEOs) targeted and inhibited major components of inflammation-related pathways, including the arachidonic acid metabolic pathway, NF-κB pathway, and MAPK pathway. We present the first study to characterize the chemical composition of BEOs and confirm their potent anti-inflammatory effects in in vitro, in vivo, and in silico analysis. These results can facilitate the development of effective anti-inflammatory drugs with limited side effects in the future.

## 1. Introduction

Inflammation is a protective response of the immune system to tissue damage or foreign stimuli, including human pathogens, allergens, toxic chemicals, or irradiation [1]. The vital functions of inflammation involve repair of damaged tissues, elimination of harmful stimuli, and maintenance of homeostasis and health [1]. However, uncontrolled inflammatory responses may lead to the initiation and progression of numerous diseases, including diabetes, Alzheimer’s disease, heart diseases, autoimmune disorders, and cancer [2,3,4]. The nonsteroidal anti-inflammatory drugs (NSAIDs) are current, widely used anti-inflammatory drugs, which inhibit the production of prostaglandins, key inflammatory mediators, by inhibiting the activity of cyclooxygenase isoenzymes (COX-1 and COX-2) [5]. As COX-1 and COX-2 are both involved in regulation of many important physiologic processes, selective or non-selective inhibition of COX-1 and COX-2 can adversely affect the stomach, kidneys, and liver [5,6,7]. Therefore, new potent anti-inflammatory drugs, especially those derived from plants with different drug targets or molecular mechanism of actions, as well as limited side effects, are urgently required to circumvent these problems [5,8].

Suitable anti-inflammatory models should be used to search for new potential anti-inflammatory drug candidates. Currently, several cell models are available for testing anti-inflammatory activities, such as RAW 264.7, THP-1, and endothelial cells [9,10]. However, the RAW 264.7 macrophages are the most commonly used cell model for assessing anti-inflammatory activities. Lipopolysaccharide (LPS) can bind to Toll-like receptor 4 (TLR4) and stimulate various inflammatory pathways in RAW 264.7 macrophages [10], including MAPK and NF-κB pathways [11,12]. The MAPK pathway—with key components such as ERK1/2, JNK, and p38—can be activated by TRAF6, which leads to the activation of AP-1 and the expression of pro-inflammatory factors [12]. The NF-κB pathway can be activated by the MyD88-dependent TLR pathway [10]. In unstimulated cells, key transcription factors of the NF-κB pathway (p50-p65) exist in a latent inactive form complexed to an inhibitor protein, IκBα. Under conditions of inflammation, IκBα is phosphorylated [13], and then degraded to induce the phosphorylation of NF-κB p65 (p-p65) [14]. The phosphorylated NF-κB p65 migrates to the nucleus and induces the expression of pro-inflammatory factors [14]. Therapeutic agents prevent inflammation by inhibiting inflammation-related signaling pathways, such as the MAPK pathway and NF-κB pathway, thereby inhibiting the production of pro-inflammatory factors, including inducible nitric oxide synthase (iNOS), cyclooxygenase 2 (COX-2), TNF-α, IL-6, NO, and PGE2.

Evaluation of bioactive compounds using animal models is important for discovering and developing new potential anti-inflammatory drugs. Different animal models are available for evaluating anti-inflammatory drugs, including chemical-induced paw edema, ear edema, vascular permeability, and pleurisy models [15,16]. Among these animal models, the carrageenan-induced edema model is widely used [17]. Inflammation induced by carrageenan is acute, local, non-immune, and reproducible in nature [15,18], which also triggers the activation of the NF-kB pathway via TLRs [19]. Involvement of multiple mechanisms of inflammation render this model suitable for preliminarily screening of anti-inflammatory drugs and assessing the anti-inflammatory activities of natural and synthetic compounds [18].

*Blumea lanceolaria (Roxb.)* Druce is a flowering plant belonging to the *Asteraceae* family, which is widely distributed across tropical and subtropical Asia, including India, China, Taiwan, Philippines, and Vietnam [20,21]. In folk medicine, *B. lanceolaria* is used to treat cancer and inflammation-associated diseases, such as fever, cough, asthma, chronic ulcers, wounds, and dysentery [20,21]. Moreover, this medicinal herb is edible and widely used in daily life for cooking fish, shrimp, pork, or snake meat [20,21]. Therefore, the chemical constituents of *B. lanceolaria* exhibit anti-inflammatory activities and are safe through oral absorption. Previous studies indicated that the methanol extracts of the leaves or roots of *B. lanceolaria* possess antioxidant and antimicrobial activities [22,23]. Using capillary gas chromatography (GC) and mass spectrometry (MS), Dung et al. (1991) found that the essential oil (EO) of *B. lanceolaria* contained mainly (95%) methyl thymol [24]. However, the chemical components of the EOs obtained from the leaves, stem, and roots of *B. lanceolaria* and their anti-inflammatory potential remain unclear. 

This study aimed to analyze in detail the chemical composition of the EOs extracted from the leaves (LBEO), stem (SBEO), and roots (RBEO) of *B. lanceolaria* plants collected in Vietnam. The anti-inflammatory activities of these EOs were analyzed using in vitro and in vivo models. The mechanism underlying the molecular interactions and affinities with key proteins in three important inflammation-related pathways, namely the arachidonic acid (AA), NF-κB, and MAPK pathways, were analyzed using molecular docking analysis. 

## 2. Results and Discussion

### 2.1. Chemical Composition of the BEOs 

**Figure 1:**
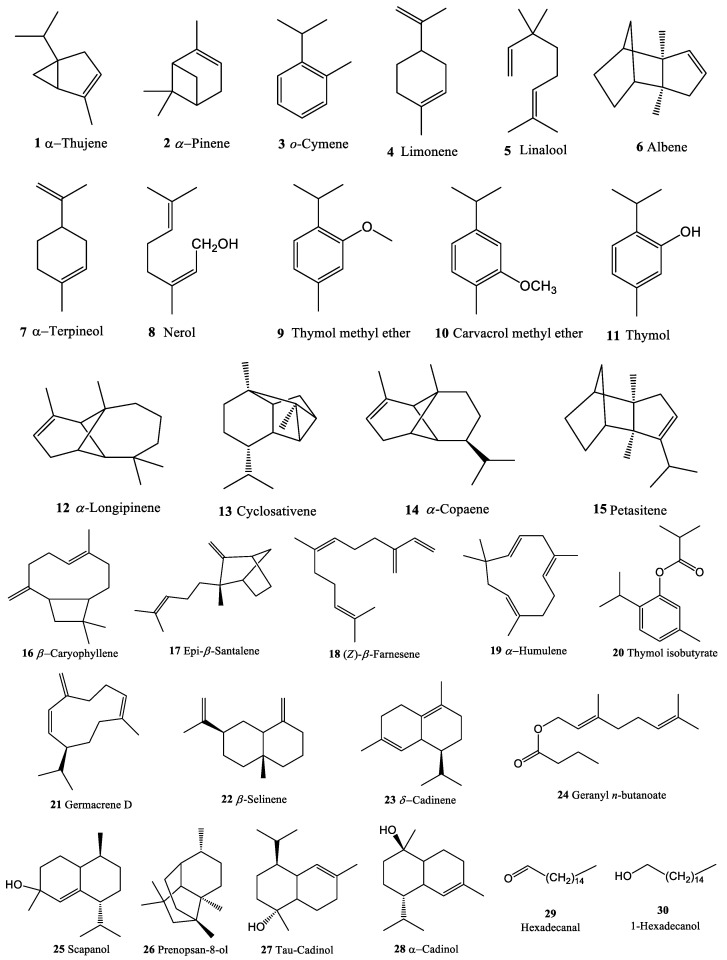
Chemical structure of the volatile compounds (**1–30**) identified from total essential oils of *B. lanceolaria*.

Table 1 shows the chemical components of LBEO, RBEO, and SBEO including the relative content (%), retention time (RT), and retention index (RI) of each constituent. In total, 30 compounds (Figure 1 and Table 1) were identified, representing 99.12%, 98.44%, and 95.62% of the total EO constituents of the leaves, stem, and roots, respectively. Among these, 5, 15, and 20 constituents were identified in the LBEO, SBEO, and RBEO, respectively, according to their mass spectra and relative RI (Table 1). 

Among the five volatile constituents of LBEO, two major compounds were identified to be *o*-cymene (38.29%) and carvacrol methyl ether (58.28%), along with three other minor constituents, namely thymol methyl ether (0.24%), *β*-caryophyllene (0.26%), and hexadecanol (2.05%). The LBEO was found to be rich in monoterpene hydrocarbons (96.81%), followed by other alcoholic compounds (2.05%) and sesquiterpenes (0.26%) (Figure S1).

Among the 15 compounds of SBEO distilled from the *B. lanceolaria* stem (Figure S2), carvacrol methyl ether accounted for 89.40% of the SBEO, followed by *o*-cymene (3.35%) and *α*-pinene (2.28%). The remaining 12 compounds were in minority, with content <1%. Monoterpenes were most abundant (95.51%), followed by sesquiterpenes (2.42%) and other compounds (0.51%).

In the EO obtained from *B. lanceolaria* root, 25 components were structurally identified using GC/MS with the HP-5 MS column. Four major compounds were found, namely, *α*-pinene (33.36%), thymol isobutyrate (29.23%), thymol methyl ether (21.70%), and thymol (4.25%). The others were minor constituents, with content <1% (Figure S3). Similar to that observed in LBEO and SBEO, RBEO contained the highest amount of monoterpenes (61.94%), followed by thymol isobutyrate (29.23%) and sesquiterpenes (4.45%).

Comparison of the major chemical components of the LBEO, SBEO, and RBEO revealed considerable variability among some of the major components in the samples. The content of carvacrol methyl ether (**10**), contributing to 58.28% of the components in LBEO, increased to 89.40% in SBEO (highest among all EOs), but disappeared in RBEO (0%). Similarly, *β*-caryophyllene (**16**) content increased from 0.26% in LBEO to 0.82% in SBEO and then decreased to 0.53% in RBEO. The contents of monoterpene *o*-cymene (from 38.29% to 3.35% and 0.92%) and hexadecanol (from 2.05% to 0.26% and 0%) tended to decrease from LBEO to SBEO and RBEO. However, *α*-pinene (**2**) was not found in LBEO, although its content increased to 2.28% and 33.36% in SBEO and RBEO, respectively. The second highest increase in content was observed for thymol methyl (**9**) ether; its content increased from 0.24% in LBEO to 0.36% in SBEO and 21.70% in RBEO. The levels of *α*-copaene (from 0% to 0.37% and 0.65%), α-humulene (from 0% to 0.17% and 0.24%), and *δ*-cadinene (from 0% to 0.18% and 0.26%) also increased. Among the 30 compounds identified from the three EO samples, three compounds (10% of the total) were only present in the stem (SBEO), including linalool (0.12%), *α*-cadinol (0.27%), and hexadecanal (0.25%). Two compounds, carvacrol methyl ether and hexadecanol, were present in the stem and leaf, but not in the root (Table 1), while fifteen compounds were found to be present only in RBEO, including thymol isobutyrate, which accounted for the greatest proportion of the components (29.23%).

The EOs obtained from the leaves and stem of *B. lanceolaria* harvested in December 2020 differed considerably from that published previously. In our plant samples, 0.24% and 0.36% thymol methyl ether (**9**) were present in the leaves and stem of *B. lanceolaria* EOs, respectively. However, Dung et al. reported that thymol methyl ether contributed to 94.96% of the content of EOs obtained from the leaves and stem of *B. lanceolaria* harvested in June 1989 [24]. The content of methyl carvacrol (**10**) was highest in our samples, with 58.28% in the leaves and 89.40% in the stem, whereas only 0.02% was reported by Dung et al. Similarly, *β*-caryophyllene (**16**) content was also high in our samples, with 0.26% in LBEO and 0.82% in SBEO, compared to 0.04% reported previously. *o*-Cymene (**3**) content was the second highest in our leaf (38.29%) and stem (3.35%) samples, although it was not reported by Dung et al. (1991). Furthermore, according to Dung et al., the contents of limonene (**4**) and α-thujene (**1**) were 0.12% and 0.04%; whereas we could not detect them in our leaf and stem samples, and only 0.14% was detected in the root sample. These differences in results can be attributed to differences in geographic factor, time of collection (December 2020 versus June 1989), and technique used (15 compounds were found in our leaf and stem samples prepared using steam distillation compared to 11 compounds reported in literature) [24]. In addition, these differences may be the result of differences in analytical method, Dung et al. used a 25 m × 0.25 mm I.D.-fused silica OV-1 (0.25 pm) column and the nitrogen gas was carried at a flow rate of 1.2 mL/min. The oven was programmed after 5 min at 60 °C, at 5 °C/min to 220 °C, with a final hold time of 20 min. In our analyses, the HP-5 MS column with a dimension of 60 m × 0.25 mm and film thickness of 0.25 μm was used for separation. Running conditions were set as follows: injector temperature at 250 °C; initial temperature started from 60 °C then increased to 240 °C with increasing step of 4 °C/min; the carrier gas was helium with the flowrate of 1 mL/min; full scan modes under electron ionization with voltage: 70 eV, emission current: 40 mA; and mass range scan: 35–450 a.m.u.

### 2.2. Effect of the BEOs on Macrophage Viability

**Figure 2:**
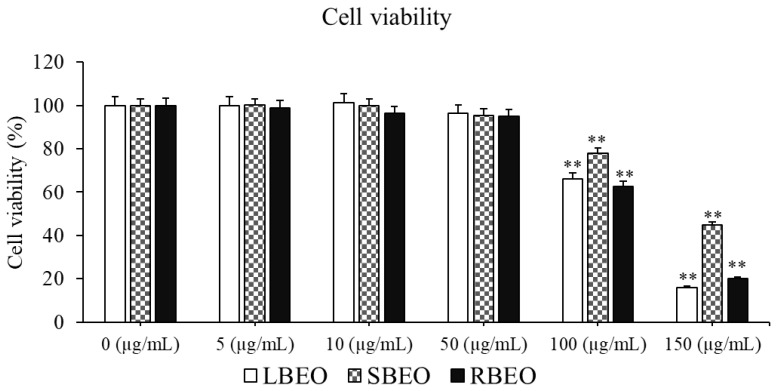
Effect of BEOs on the viability of RAW 264.7 macrophages. Various concentrations of LBEO, SBEO, and RBEO (0, 5, 10, 50, 100, and 150 µg/mL) were added to macrophage cell cultures for 24 h at 37 °C, the cell viability was then assessed using the CCK-8 kit. Data from three independent experiments were used to calculate mean values and standard deviation. ** *p* < 0.001 vs. negative control (0 µg/mL EOs).

We evaluated the toxicity of BEOs in RAW 264.7 using the CCK-8 kit (Abcam, Cambridge, UK). The results indicate that at concentrations of 0–50 µg/mL, none of the three essential oil samples were toxic to the RAW 264.7 cells (Figure 2). However, 100 µg/mL LBEO, SBEO, and RBEO were cytotoxic, causing 34.89%, 22.12%, and 37.28% cell death, respectively. When the concentration of the BEOs was increased to 150 µg/mL, the cell survival rate decreased to <50%. Thus, 50 µg/mL BEO is relatively safe for cells, with a survival rate of more than 95%. The calculated IC_50_ values of LBEO, SBEO, and RBEO are 151.11, 335.60, and 123.05 µg/mL, respectively. Therefore, the optimal concentration range of 5–50 µg/mL, at which the EOs were not toxic but active, was selected for anti-inflammatory studies.

### 2.3. Inhibition of NO Production by BEOs

**Figure 3:**
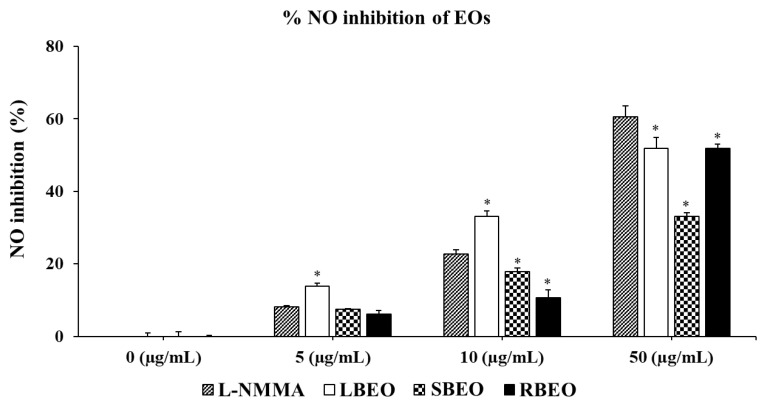
Effects of LBEO, SBEO, and RBEO on NO production in RAW 264.7 macrophages. NO secretion in the macrophages treated with 5, 10, and 50 µg/mL BEOs was measured using the Griess reagent system. Data from three independent experiments were used to calculate mean values and standard deviation * *p* < 0.01 vs. positive control group (L-NMMA).

Inhibitory effects of BEOs on NO production in RAW 264.7 cells were assessed using the Griess reagent (Promega, Madison, WI, USA). As shown in Figure 3, all three EOs (BLEO, SBEO, and RBEO) remarkably inhibited NO production (*p* < 0.05) in a concentration-dependent manner. Among the EOs, LBEO showed the best NO inhibition ability at all the three tested concentrations (5, 10, and 50 µg /mL). Both 50 µg/mL LBEO and RBEO showed the highest NO inhibition ability (approximately 50%). Compared to LBEO and RBEO, 50 µg/mL SBEO showed the lowest NO inhibitory activity of 33.15 ± 1.01%. Thus, all three EOs markedly inhibited NO production in RAW 264.7 macrophages.

### 2.4. Effect of the BEOs on Transcription and Translation of TNF-α and IL-6

**Figure 4:**
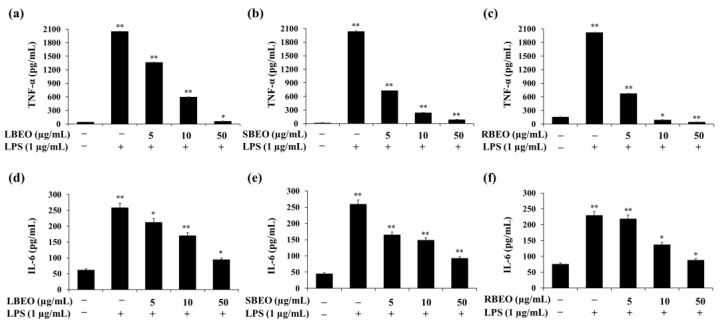
Inhibitory effects of LBEO, SBEO, and RBEO (5, 10, and 50 µg/mL) on TNF-α and IL-6 production in RAW 264.7 macrophages. After 24 h of stimulation by LPS, the inhibition of TNF-α and IL-6 production by (**a**,**d**) LBEO, (**b**,**e**) SBEO, and (**c**,**f**) RBEO were quantified by ELISA. Cytokine concentrations were calculated using a standard curve. Data from three independent experiments were used to calculate mean values and standard deviation. * *p* < 0.05, ** *p* < 0.01 vs. the untreated group.

The ability to inhibit the production of TNF-α and IL-6 is an important indicator for evaluating the anti-inflammatory property of BEOs. ELISA results (Figure 4) show that the level of TNF-α increased to more than 2000 pg/mL when stimulated by LPS. TNF-α production decreased from 2000 pg/mL to 61, 91, and 44 pg/mL in cells treated with 50 µg/mL BLEO, SBEO, and RBEO, respectively. These results indicate that all three BEOs strongly inhibit TNF-α production. Similarly, in the presence of BEOs, the level of IL-6 decreased significantly compared to that in the control sample (LPS-treated macrophages). The amount of IL-6 decreased from 250 pg/mL to 94.67, 92.67, and 88 pg/mL when treated with increasing concentrations (up to 50 µg/mL) of LBEO, SBEO, and RBEO, respectively (Figure 4d–f).

**Figure 5:**
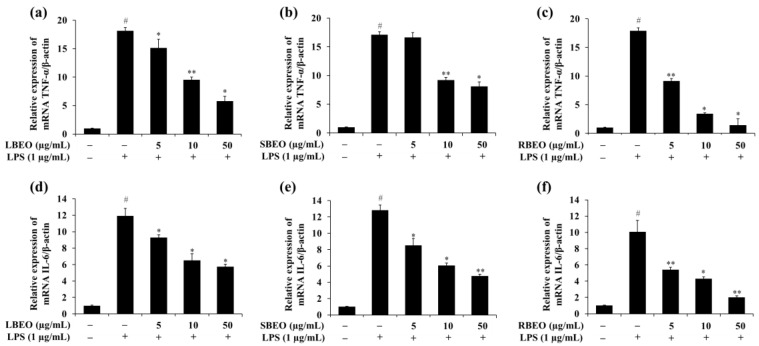
Effect of BEOs on the TNF-α and IL-6 mRNA levels of macrophages. Effects of (**a**) BLEO, (**b**) SBEO, and (**c**) RBEO on the TNF-α mRNA expression and effects of (**d**) BLEO, (**e**) SBEO, and (**f**) RBEO on the IL-6 mRNA expression in RAW 264.7 cell model after LPS stimulation were analyzed by qRT-PCR. The β-actin mRNA level was used as the interior standard to normalize the data, and the results were compared with those for the untreated cells. Data from three independent experiments were used to calculate mean values and standard deviation; # *p* < 0.05 vs. the LPS-untreated cells group, * *p* < 0.05, and ** *p* < 0.01 vs. the LPS-treated group.

The expression of the TNF-α mRNA in LPS-treated cells (Figure 5a–c) was approximately 17.71-fold higher than that in cells not stimulated by LPS; this decreased to 5.76-, 8.09-, and 1.44-fold when the cells were incubated with 50 µg/mL of LBEO, SBEO, and RBEO, respectively. The IL-6 mRNA level increased by approximately 11.6-fold in the LPS-supplemented samples, which decreased to 5.73-, 4.75-, and 2.02-fold in the presence of 50 µg/mL of LBEO, SBEO, and RBEO, respectively (Figure 5d–f). These results suggest that LBEO, SBEO, and RBEO acted as anti-inflammatory agents in LPS-induced RAW 264.7 cells by suppressing the production of TNF-α and IL-6. Among the three EOs, RBEO inhibited the transcription and translation of the pro-inflammatory cytokines the most (Figure 4 and Figure 5).

### 2.5. Effect of the BEOs on the Transcription and Translation of iNOS and COX-2

**Figure 6:**
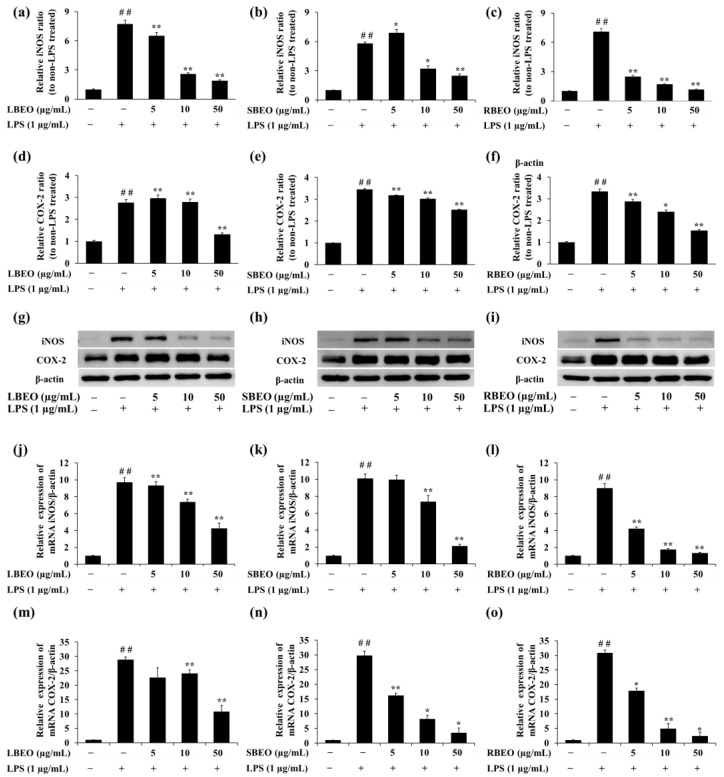
Inhibitory effects of BEOs on iNOS and COX-2 expression in RAW 264.7 macrophages. The iNOS and COX-2 protein levels in (**a**,**d**,**g**) LBEO-, (**b**,**e**,**h**) SBEO-, and (**c**,**f**,**i**) RBEO-pre-treated cells were assessed using Western blotting after 24 h LPS induction. The iNOS and COX-2 mRNA expression levels in (**j**,**m**) LBEO-, (**k**,**n**) SBEO-, and (**l**,**o**) RBEO-pre-treated RAW 264.7 cells were quantified using qRT-PCR after 24 h LPS induction. The β-actin mRNA level was used to normalize the data. The results were compared to those in the untreated cells. Results are shown as mean ± SD (*n* = 3). ## *p* < 0.01 vs. the LPS-untreated cells; * *p* < 0.05, ** *p* < 0.01 vs. the LPS alone-treated cells.

COX-2 and iNOS are important pro-inflammatory proteins responsible for the synthesis of NO and PGE2. Western blotting and qRT-PCRwere performed to evaluate whether the EOs inhibited expression of iNOS and COX-2 at both transcription (mRNA) and translation (protein) levels in the RAW 264.7 cell model. Corresponding to the results of NO production mentioned previously (Figure 3), the expression levels of iNOS at transcription level (Figure 6j–l) and translation level (Figure 6a–c,g–i) were significantly inhibited in the presence of BEOs. The inhibition of the iNOS expression (both mRNA and protein levels) was most effective in the RBEO sample (Figure 6c,i,l) where the mRNA and protein levels decreased to the levels observed in LPS-untreated cells.

COX-2 is an important enzyme that controls the production of PGE2 in the inflammatory response [25,26]. Figure 6 shows the effect of BEOs on COX-2 expression at both transcription and translation levels. The COX-2 protein expression increased significantly in response to LPS stimulation. However, in the presence of BEOs (5, 10, or 50 μg/mL), the COX-2 protein expression decreased in a concentration-dependent manner (Figure 6d–f). Moreover, COX-2 mRNA levels (Figure 6m–o) also decreased in the presence of BLEO, SBEO, and RBEO. Thus, BEOs can inhibit COX-2 expression at mRNA and protein levels.

### 2.6. Inhibitory Effect of BEOs on the NF-κB Pathway in RAW 264.7 Macrophages

**Figure 7:**
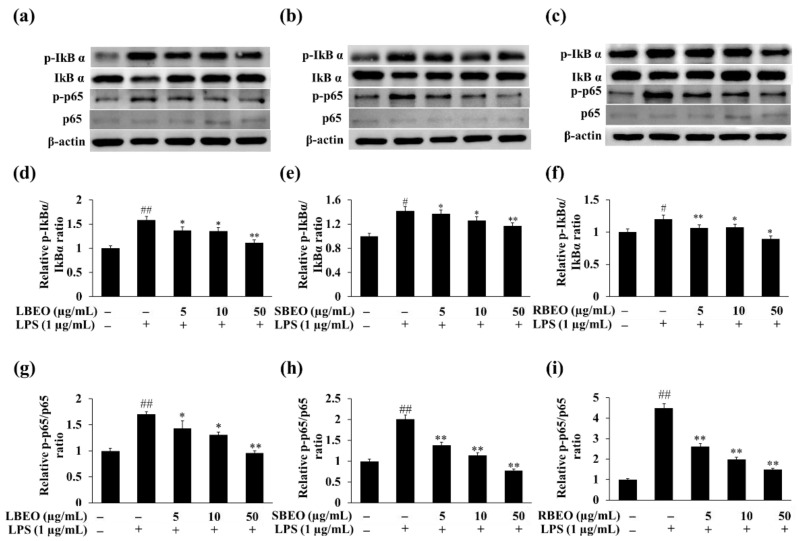
Effects of LBEO, SBEO, and RBEO on protein levels of IκBα, p-IκBα, p65, and p-p65 in RAW 264.7 macrophages. Western blot analysis of non-phosphorylated and phosphorylated forms of IκBα and p65 in LPS-induced macrophages treated with (**a**) LBEO, (**b**) SBEO, and (**c**) RBEO. Relative expression level of p-IκBα and p-p65 in (**d**,**g**) LBEO-, (**e**,**h**) SBEO-, and (**f**,**i**) RBEO-treated group compared to non-stimulated group. The results are expressed relative to those for the LPS-untreated cells as mean ± SD (*n* = 3). # *p* < 0.05, and ## *p* < 0.01 vs. the LPS-untreated cells; * *p* < 0.05, and ** *p* < 0.01 relative to the LPS alone-treated cells.

The NF-κB pathway regulates many genes involved in the inflammatory response [15,27,28]. To further investigate the inhibitory targets of EOs, we analyzed whether BEOs can affect the phosphorylation of two key proteins (IkBα and p65) in NF-κB pathway by monitoring the levels of IκBα, phosphorylated IκBα (p-IκBα), p65, and phosphorylated p65 (p-p65) in RAW 264.7 macrophages using Western blotting. In the presence of increasing concentrations of LBEO, SBEO, and RBEO (Figure 7a–f), the level of phosphorylated IκBα in LPS-stimulated macrophages decreased gradually to that observed in the unstimulated stage. The levels of p-p65 gradually decreased to the level in the unstimulated state when the EO concentration reached 50 µg/mL (Figure 7a–c,g–i). These results suggest that BEOs inhibited the NF-κB pathway by inhibiting p65 and IκBα phosphorylation.

### 2.7. In Vivo Anti-Inflammatory Assay

**Figure 8:**
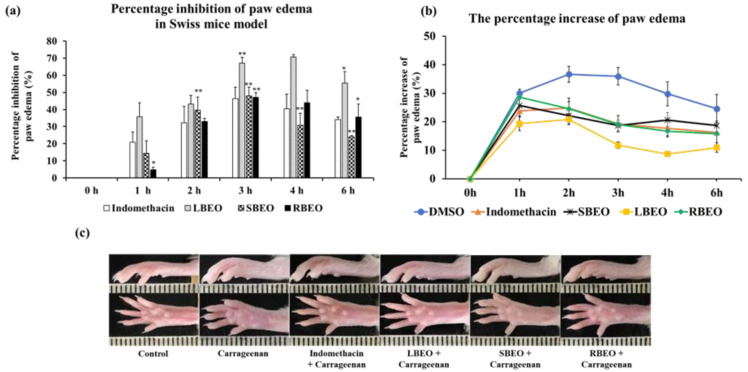
Inhibition of paw edema in mice by LBEO, SBEO, and RBEO. (**a**) The percentage inhibition of paw edema in mice over time was monitored after treatment with LBEO, SBEO, RBEO, and the positive control (indomethacin). Data are shown as mean ± SD (*n* = 6 mice/group), * (*p* < 0.01), and ** (*p* < 0.001) vs. indomethacin group. (**b**) The percentage increase in paw edema. (**c**) Image of hind paws of mice at 3 h after carrageenan injection.

To evaluate the anti-inflammatory effect of BEOs at the in vivo level, we used Swiss mice as an animal model for the carrageenan-induced paw edema test. As shown in Figure 8a, all the EOs (LBEO, SBEO, and RBEO) inhibited paw edema in the Swiss mice model compared to that observed after treatment with indomethacin, a common nonsteroidal anti-inflammatory drug. Among the four samples (LBEO, SBEO, RBEO, and indomethacin), LBEO showed the maximum inhibition of paw edema throughout the trial period of 1–6 h after carrageenan-induced inflammation, with percentage inhibition of paw edema being 35.66 ± 8.29%, 43.11 ± 5.12%, 67.22 ± 3.31%, 70.74 ± 1.40%, and 55.45 ± 6.61% after 1, 2, 3, 4, and 6 h, respectively. The edema inhibition abilities of SBEO and RBEO were similar to that of indomethacin from 2 to 6 h after carrageenan induction. Compared to the other time points, at about 3 h after carrageenan induction, all three essential oils showed the highest inhibition (*p* < 0.001) of paw edema in mice; LBEO showed 67.22 ± 3.31% inhibition, while, LBEO, SBEO, and the positive control (indomethacin) showed 48.03 ± 5.05%, 47.01 ± 2.87%, and 46.38 ± 6.59% inhibition, respectively. The percentage increase in paw edema in Figure 8b and the images of the mouse paw edema (Figure 8c) at 3 h also indicated that the inhibitory effects of LBEO, SBEO, and RBEO on paw edema were similar or even better than that of indomethacin.

Carrageenan-induced paw edema model is a well-known in vivo model for studying acute inflammation. In this model, carrageenan induces edema in two-phase responses, namely, the early and delayed phases. The early phase (0−2 h) involves the production of histamines, serotonin, and bradykinin, while the delayed phase (3−6 h) involved the release of NO and pro-inflammatory cytokines, such as IL-6 and TNF-α. Our in vivo results show that all three EOs inhibited paw edema at both phases. This agrees with our in vitro results showing that LBEO, SBEO, and RBEO can inhibit multiple steps in the inflammatory responses of the RAW 264.7 cell model, including (1) NO production; (2) TNF-α, IL-6, iNOS, and COX-2 expression at both mRNA and protein levels; and (3) IκBα and p65 phosphorylation in the NF-κB pathway.

### 2.8. Molecular Docking of 12 Main Compounds with Anti-Inflammatory Protein Targets

We showed that BEOs possessed anti-inflammatory activities and also revealed some of the important targets of these activities using Western blotting, ELISA, and RT-PCR. However, the underlying molecular mechanisms and interaction affinities of the EO components with inflammatory protein targets remain unknown. Hence, to further investigate the potential interaction affinity of BEO compounds with inflammatory protein targets, we selected 12 main compounds (Table 2) present in BEOs, which were then docked with inflammatory protein targets [28,29,30] of the AA, NF-κB, and MAPK pathways. The results of molecular docking are summarized in Table 2.

#### 2.8.1. Validation of the Molecular Docking Method

**Figure 9:**
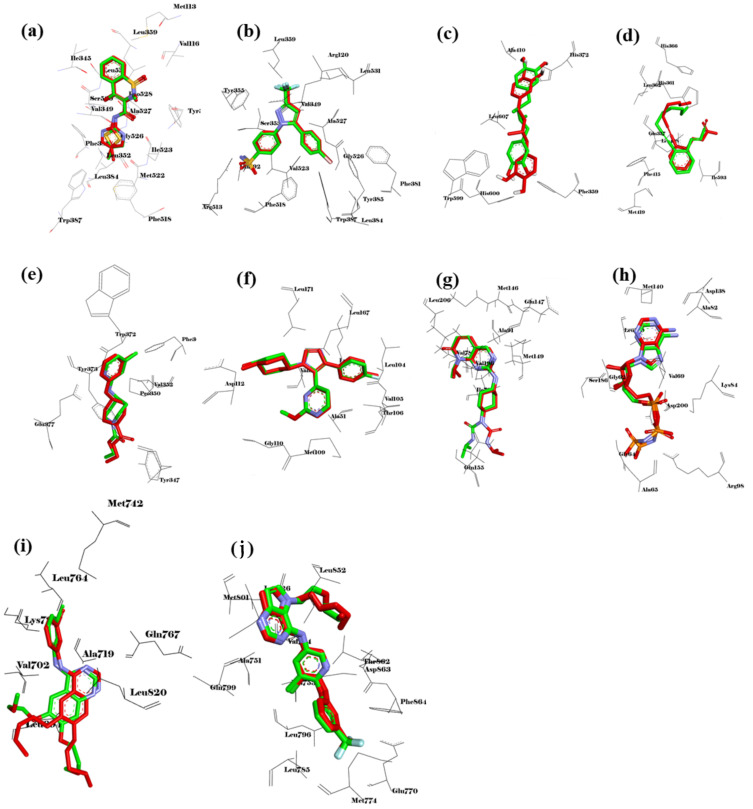
Validation of the molecular docking method. Overlay of redocked conformation (green carbons) with co-crystalized conformation (red carbons) of (**a**) meloxicam to COX-1 (4O1Z), (**b**) celecoxib to COX-2 (6COX), (**c**) 30Z to 5-LOX (6N2W), (**d**) RS7 to ALOX15 (2P0M), (**e**) AT2 to iNOS (3E7G), (**f**) SB0 to p38MAPK (4FA2), (**g**) PDB ID:519 to JNK (4Y5H), (**h**) ANP to ERK5 (4IC7), (**i**) AQ4 to EGFR (1M17), and (**j**) 03Q to ERBB2 (3PP0).

To obtain reliable results in molecular docking analysis, the molecular docking method should be validated. Toward this, reference ligands were obtained from native co-crystal structures, including meloxicam for COX-1 (PDB ID: 4O1Z), celecoxib for COX-2 (PDB ID: 6COX), 30Z for 5-LOX (PDB ID: 6N2W), RS7 for ALOX15 (PDB ID: 2P0M), AT2 for iNOS (PDB ID: 3E7G), SB0 for p38MAPK (PDB ID: 4FA2), ligand PDB ID:519 for JNK (PDB ID: 4Y5H), ANP for ERK5 (PDB ID: 4IC7), AQ4 (erlotinib) for EGFR (PDB ID: 1M17), and 03Q for ERBB2 (PDB ID: 3PP0). All the reference ligands docked back well to the target protein structures (Figure 9). The re-docked conformations overlaid well with their crystal conformations, with the RMSD values ranging from 0.049 Å (COX-1) to 1566 Å (5-LOX). These results indicate that our docking method provided reliable results and can be used to further analyze the interaction of other potential ligands with the target protein structures.

#### 2.8.2. AA Metabolic Pathway

**Figure 10:**
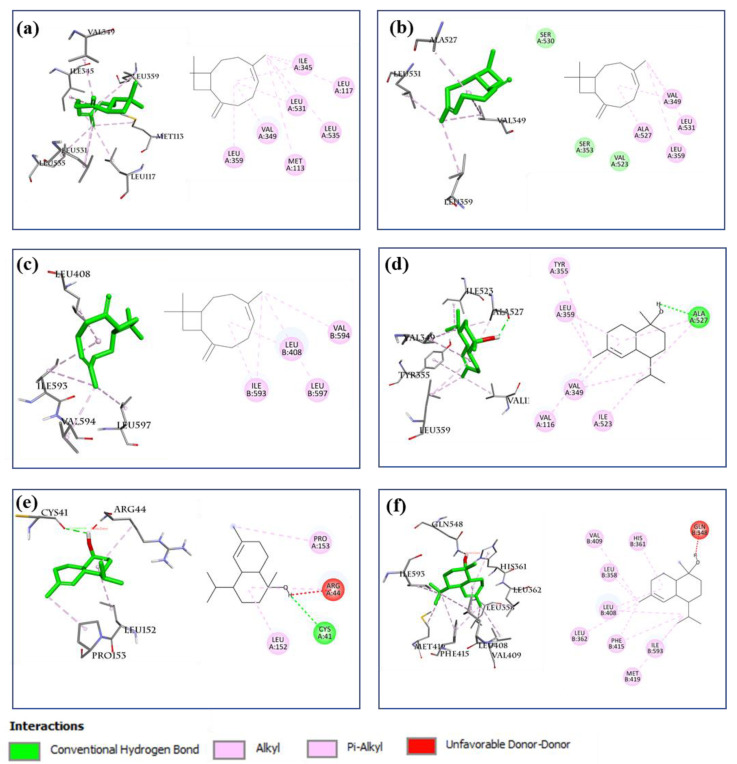
Docking analysis of compounds 16 and 27 with COX-1, COX-2, and ALOX15. Interaction analysis of compound **16** (left: 3D, right: 2D) with (**a**) COX-1 (PDB ID: 4O1Z), (**b**) COX-2 (PDB ID: 6COX), and (**c**) ALOX15 (PDB ID: 2P0M). Interaction analysis of compound **27** (left: 3D, right: 2D) with (**d**) COX-1(PDB ID: 4O1Z), (**e**) COX-2(PDB ID: 6COX), and (**f**) ALOX15 (PDB ID: 2P0M).

The AA metabolic pathway is associated with the development and elimination of inflammation [15,28,31,32]. Key proteins associated with inflammatory responses of the AA pathway, including COX-1, COX-2, 5-LOX, and ALOX15, were selected to evaluate their interaction with 12 selected compounds present in the BEOs. The docking results (Table 2) show that all 12 compounds (except compound **30**) showed good binding affinity for COX-1, COX-2, and ALOX15, with binding values below −6.0 kcal/mol. The lower the calculated binding values, the better the binding ability. Thus, compounds **14**, **16**, **19**, **20**, **23**, and **27** showed extremely strong binding affinity for COX-1, COX-2, and ALOX15, with binding values lower than −7.0 kcal/mol. All the compounds showed weaker binding with 5-LOX, among which, the binding affinities of compounds **14**, **16**, **19**, **20**, **23**, and **27** ranged from −6.1 to 6.4 kcal/mol. These results suggest that the main compounds of BEOs can target and interact with multiple targets of the AA pathway. In particular, compound **16** (β-caryophyllene), present in all three types of EOs (leaf, stem, and root), showed considerably high binding affinity for COX-1 (−9.0 kcal/mol), COX-2 (−8.1 kcal/mol), and ALOX15 (−8.2 kcal/mol). This partially explained why all three types of BEOs showed good anti-inflammatory effects, although their compound compositions may differ. Compound **27** (present in SBEO and RBEO) also showed very strong binding with COX-1 (−7.0 kcal/mol), COX-2 (−7.8 kcal/mol), 5-LOX (−6.4 kcal/mol), and especially ALONX15 (−9.1 kcal/mol). The binding locations of **16** and **27** with COX-1, COX-2, and ALOX15 (Figure 10) are similar to the binding locations of the reference co-crystal ligands (Figure 9), indicating the reliability of the docking method. These results show that the bioactive compounds of the BEOs can target key components of the AA pathway, thereby inhibiting inflammatory responses.

#### 2.8.3. NF-κB Signaling Pathway

**Figure 11:**
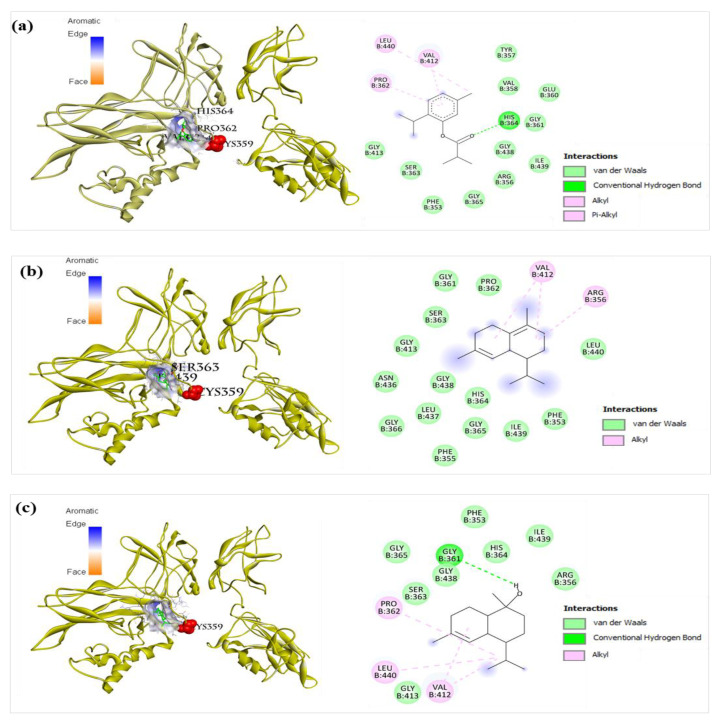
Docking analysis of compound **20**, **23**, and **27** with NF-κB p50-p65. (**a**) Compound **20** (left: 3D, right: 2D) showed pi-alkyl interaction with Val412, alkyl interaction with Pro362 and Leu440, and hydrogen bonding with His364 of NF-κB (PDB ID: 1VKX). (**b**) Compound **23** (left: 3D, right: 2D) showed alkyl interaction with Val412 and Arg356, and van der Waals interactions with Phe353, Phe355, Gly361, Pro362, Ser363, His364, Gly366, Gly413, Gly365, Asn436, Leu437, Gly438, Ile439, and Leu440, of NF-κB (PDB ID: 1VKX). (**c**) Compound **27** (left: 3D, right: 2D) showed alkyl interaction with Pro362, Val412, and Leu440, and hydrogen bonding with Gly361 residues of p50 (PDB ID: 1VKX).

The NF-κB pathway is also associated with inflammation [15,27,28]. Therefore, important components of the NF-κB signaling pathway (TLR, NF-κB p50p65, TNF-α, and iNOS) were selected for studying their interactions with components of BEOs using molecular docking analysis. No co-crystal structure of inhibitor/NF-κB complex is reported so far. Therefore, the re-dock strategy cannot be used to validate docking methods for NF-κB. However, a docking method that was confirmed to be effective for NF-κB proteins in previous studies [33,34] can be used, in which the binding region of NF-κB with DNA fragment was used as the binding site for docking. The grid box was set at 50 × 50 × 50 Å with a 0.375 Å space to cover the DNA binding pocket of the most prevalent form of NF-κB, p50-p60 (PDB ID: 1VKX), and all other docking parameters were set to default values. The docking results (Table 2) show that compounds **10**, **11**, and **16** have good binding affinity for the NF-κB p50-p65 homodimer complex, with the calculated binding values of −6.2, −6.1, and −6.7 kcal/mol, respectively. Compounds **20**, **23**, and **27** showed strong binding affinities for NF-κB p50-p65, with the calculated binding values of −7.0, −7.6, and −7.1 kcal/mol, respectively. Interestingly, these compounds interacted with the cavity near the cysteine 359 of p50 (Figure 11a–c). This result agrees with that of a previous study showing that the binding region of the p50 inhibitor (andrographolide) is near the cysteine 359 of p50 [35]. This suggests that many compounds of BEOs can interact and inhibit NF-κB p50.

Another important component of the NF-κB pathway is iNOS. Compounds **10**, **11**, **14**, **16**, **19**, **20**, **23,** and **27** showed good interaction with iNOS, with binding affinity values lower than -6.0 kcal/mol. Compounds **14**, **16**, **19**, **20**, **23**, and **27** also showed good interaction with TNF-α, with binding affinity values lower than -6.0 kcal/mol. However, TLR did not appear to be the inhibitory target of BEO components when all the calculated binding values ranged from -3.1 to -5.2 kcal/mol (Table 2). These results suggest that NF-κB, TNF-α, and iNOS are the main targets of the bioactive compounds in BEOs.

#### 2.8.4. MAPK Signaling Pathway

**Figure 12:**
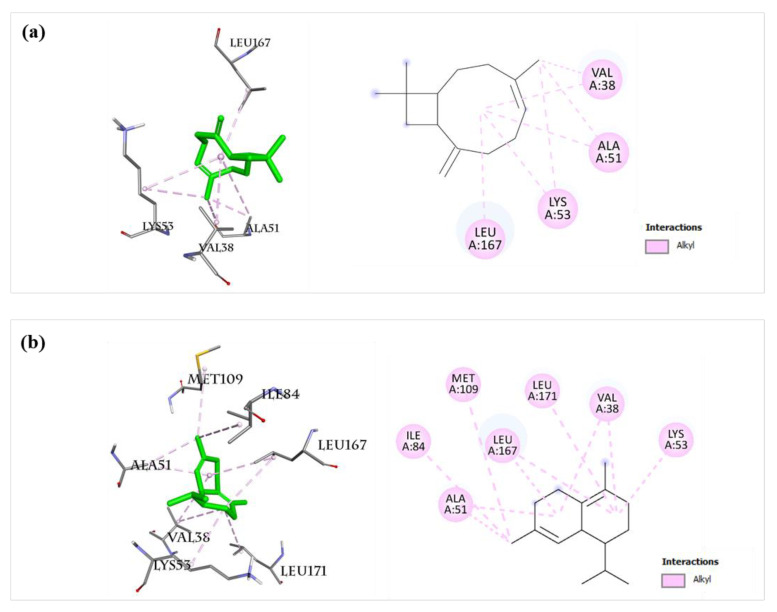
Docking analysis of compounds **16** and **23** with p38MAPK. (a) Compound **16** (left: 3D, right: 2D) showed alkyl interaction with Val38, Ala51, Lys53, and Leu167 residues of p38MAPK (PDB ID: 4FA2). (**b**) Compound **23** (left: 3D, right: 2D) showed alkyl interaction with Val38, Ala51, Lys53, Ile84, Met109, Leu167, and Leu171 residues of p38MAPK (PDB ID: 4FA2).

The MAPK signaling pathway is one of the key pathways for inducing inflammatory responses [15,27,28]. In this study, important components of the MAPK pathway mediating inflammatory responses, including JNK, p38 MAPK, ERK5, EGFR [36], and ERBB2 [37], were selected for the docking study with the twelve main components of BEOs. The docking results (Table 2) suggest that almost all compounds (except for compound **30**) docked well with five target proteins of the MAPK pathway, with calculated binding affinities lower than −6.0 kcal/mol. Compounds **14**, **16**, **19**, **23**, and **27** showed strong binding affinities for all the five target proteins (p38MAPK, JNK, ERK5, EGFR, and ERBB2), as their calculated binding affinities were lower than -7.0 kcal/mol. Compounds **16** and **23** showed strong binding ability with p38 MAPK, with the binding affinity values of −8.2 and −8.5 kcal/mol, respectively, which are better than that of a reference compound (SB0) (−7.8 kcal/mol) for p38 MAPK. The binding sites of compounds **16** and **23** with p38 MAPK (Figure 12) are similar to that of SB0 (Figure 9), indicating the reliability of our docking method. Compound **16** showed very high binding with p38MAPK, JNK, ERK5, and ERBB2, with calculated binding affinity values of −8.2, −8.0, −7.2, and −7.7 kcal/mol, respectively. These results suggest that many bioactive compounds of BEOs can interact and inhibit multiple important targets of the MAPK pathway.

#### 2.8.5. Inflammatory Interleukins (IL-6 and IL-23R)

Inflammatory interleukins also play essential roles in inflammatory responses [29,38]. In this study, IL-6 and IL-23 receptors (IL-23R) were selected for molecular docking studies. Almost all the 12 selected compounds showed low binding affinity for IL-6 and IL-23R (ranging from −3.6 to 5.9). Only compound **27** showed good binding affinity for IL-6 and IL-23R, with binding values of −6.2, and −6.1 kcal/mol, respectively. Compound **16** showed good binding affinity for IL-6, with a binding affinity value of −6.1 kcal/mol. Overall, the binding abilities of the selected compounds of BEOs with inflammatory cytokines (IL-6, IL-23R) were not as high as those of other inflammatory targets, such as COX-1, COX-2, ALONX15, NF-κB, p38MAPK, JNK, and ERBB2. These results suggest that IL-6 and IL-23R are not the key anti-inflammatory targets of BEOs.

Although the 12 main compounds of BEOs have good docking scores with important anti-inflammatory protein targets, the anti-inflammatory effects of BEOs may also be due to interactions with other inflammatory protein targets. The in silico anti-inflammatory effects of the 18 other compounds of low abundance in the BEOs (especially RBEO) have to be analyzed using molecular docking in the future.

## 3. Materials and Methods

### 3.1. Experimental Animals and Ethics Statement

Swiss mice (female, 8–12-week-old, 20–25 g) from the National Institute of Hygiene and Epidemiology, Hanoi, Vietnam were maintained at the Animal Facility, Faculty of Biology, HUS, Vietnam National University, Vietnam, using standard conditions with commercial food and water ad libitum. Ethical approval for animal studies was obtained from the Ethic Committee of the Dinh Tien Hoang Institute of Medicine (certificate number: IRB-A-2102).

### 3.2. Plant Material

The whole plant, containing leaves, stem, and roots of *B. lanceolaria* were collected from Hanoi city (21°2′46.46′′ N, 105°24′56.59′′ E), Vietnam, in December 2020. The plant samples were identified and deposited at the Center of Life Science, Faculty of Biology, University of Science, Vietnam National University, Hanoi, Vietnam, under specimen vouchers (No. 20201201, No. 20201202, and No. 20201203).

### 3.3. Preparation of EOs

The plant materials were separated into three parts: leaves, stem, and roots. These materials were then sliced into small pieces before being steam-distilled to obtain three essential oils, namely, LBEO (0.1677%), SBEO (0.0111%), and RBEO (0.0177%).

### 3.4. GC/MS Analyses

The GC-MS/GC-flame ionization detection (FID) comprising of an HP7890A model GC and HP5975C MS detector (Agilent Technologies, Santa Clara, CA, USA) was used for analyzing the chemical constituents of LBEO, SBEO, and RBEO. A HP-5 MS column with a dimension of 60 m × 0.25 mm and film thickness of 0.25 μm was used for separation. The running condition was set as follows: injector temperature at 250 °C; initial temperature started at 60 °C then increased to 240 °C with an increasing step of 4 °C/min; the carrier gas was used of helium and the flowrate was set as 1 mL/min; the split ratio was 100:1; full scan modes under electron ionization with voltage: 70 eV, emission current: 40 mA; mass range scan: 35–450 a.m.u. Chemical constituents in each essential oil were identified by analysis of RI and MS values in comparing with standard compounds in the NIST database and literature [39].

### 3.5. Chemicals and Reagents

*Escherichia coli* LPS, and λ-carrageenan were obtained from Sigma (St. Louis, MO, USA). Primary antibodies (Anti-iNOS, anti-COX2, anti-IκBα, anti-p-IκBα, anti-β-actin antibodies) and secondary antibody (goat anti-rabbit IgG (H + L), HRP) were purchased from Invitrogen (Rockford, IL, USA). Anti-p65 and anti-phospho- p65 were obtained from Bio-Rad (Oxford, UK).

### 3.6. Cell Culture

The RAW 264.7 cells were obtained from the Animal Cell Culture Lab, Faculty of Biology, University of Science, Vietnam National University. The cells were grown in DMEM medium at 37 °C with 10% FBS, 50 µg/mL streptomycin/penicillin, and 5% CO_2_.

### 3.7. Cell Viability Assay

The toxicity of the EO samples toward RAW 246.7 macrophages was determined using CCK-8 (ab228554, Abcam, Cambridge, UK). The macrophages were maintained at 37 °C for 24 h in a 96-well plate (2 × 10^4^ cells in 100 µL culture per well) before being treated with various concentrations of EOs from *B. lanceolaria* (BEOs) for another 24 h. Each sample was incubated with 10 μL of CCK-8 solution for 2 h at 37 °C before being measured at the OD of 450 nm.

### 3.8. Measuring NO Production

RAW 264.7 macrophages (2 × 10^5^ cells/well) were cultured in FBS-free DMEM for 3 h before being incubated with EOs (0, 5, 10, and 50 µg/mL) for 2 h. The cells were then incubated with LPS (1 μg/mL) for 24 h to stimulate NO production. NG-methyl-L-arginine acetate (L-NMMA) was used as the positive control. Cell culture medium (100 μL) was mixed with 100 μL Griess reagent (Promega, Madison, WI, USA) at 25 °C for 10 min and the absorbance at 540 nm was then recorded using the SpectraMax Plus^384^ microplate reader (Molecular Devices, California, USA).

The inhibition of NO production was determined using the formula:

$$\%\mathrm{Inhibition}=100-\frac{\left[\mathrm{NO}\right]\mathrm{sample}}{\left[\mathrm{NO}\right]\mathrm{LPS}}\times 100.$$

### 3.9. ELISA

The macrophages (2 × 10^5^ cells/well) were maintained for 24 h at 37 °C before being treated to 5, 10, and 50 µg/mL BEOs for 2 h. The cells were then stimulated by LPS (1 µg/mL) for 24 h. The supernatant was used to measure the concentrations of TNF-α and IL-6 produced using ELISA kits (Invitrogen, Vienna, Austria).

### 3.10. Western Blotting

The effects of BEOs on the expression of important proteins involved in the inflammatory response in RAW 264.7 macrophages were assessed by Western blotting. The cells (4 × 10^5^ cells/well in 6-wells plate) were cultured for 24 h before incubating with BEOs for 2 h. The cells were then stimulated by LPS (1 µg/mL) for 24 h before being harvested and resuspended in radioimmunoprecipitation assay lysis buffer (Thermo Scientific, Rockford, IL, USA) to obtain the total protein. Total protein samples (20 µg each) were analyzed using SDS-PAGE before being transferred to a PVDF membrane. The membrane was blocked with T-TBS (Tris-buffered saline, 0.1% Tween 20) containing 2% bovine serum albumin (Biobasic, Markham, Ontario, Canada) for 1 h. The membrane was then incubated with primary antibodies (1:1000 dilutions) for detecting β-actin, iNOS, COX-2, IκBα, p-IκBα, NF-κB p65, and p-NF-κBp65 for 16 h at 4 °C. The membranes were then washed thrice with T-TBS before being incubated with HRP-conjugated goat anti-rabbit IgG for 1 h at 25 °C. The membranes were washed thrice and then incubated with Clarity Max^TM^ Western ECL substrate (Bio-Rad, Milan, Italy) and the signals were detected using a ChemmiDoc^TM^ imaging system (Bio-Rad, Hercules, CA, USA). Protein quantities from Western blot results were calculated by Image Lab software Version 6.1 (Bio-Rad, Hercules, CA, USA).

### 3.11. qRT-PCR

To determine the effect of BEOs on the mRNA production of IL-6, TNF-α, iNOS, and COX-2, the RAW 264.7 macrophages (4 × 10^5^ cells/well) were cultured for 24 h before being treated with LBEO, SBEO, and RBEO (5, 10, and 50 µg/mL) for 2 h. Inflammatory responses were stimulated by LPS (1 µg/mL) for 16 h. Total RNA (5 µg/mL) purified from each treated sample using the TRIzol™ reagent (Invitrogen, Rockford, Illinois, USA) was converted to cDNA using M-MLV reverse transcriptase (Thermo Fisher Scientific). The qPCR reaction (10 µL in total volume) contained 2 µL cDNA templates, 5 µL GoTaq^®^ qPCR master mix (Promega, Madison, WI, USA), 0.25 µL forward/reverse primers (Table 3), and 2.5 µL H_2_O. All reactions were run with 40 cycles; each cycle included DNA denaturation step at 95 °C for 30 s, primer annealing step at 60 °C for 30 s, and DNA strand extension step at 72 °C for 30 s. Each reaction was run in triplicate. The relative mRNA levels were calculated using the 2^-∆∆Ct^ method [40]; β-actin mRNA level was used as an internal standard for normalizing expression data.

### 3.12. Anti-Inflammation Assay Using Carrageenan-Induced Edema Model

The effects of BEOs on acute inflammation in an animal model were evaluated using the carrageenan-induced paw edema model [44]. Swiss mice (21–24 g) were stabilized under laboratory conditions for at least 7 days prior to testing. Five groups of mice (*n* = 6 mice/group) were prepared, including: (1) negative control group (dimethyl sulfoxide or DMSO, 10 mL/kg), (2) positive control group (indomethacin, 10 mg/kg), (3) LBEO (50 mg/kg), (4) SBEO (50 mg/kg), and (5) RBEO (50 mg/kg). Mice were orally administered with indomethacin, or BEOs, and DMSO (the vehicle control). After 60 min, 0.025 mL of 2% carrageenan suspension (Sigma-Aldrich) was injected under the soles of the right hind paws to induce inflammation. The changes in the right hind paw thickness of the mice at 0, 1, 2, 3, 4, and 6 h after carrageenan injection was measured using a micrometer (Mitutoyo, Japan). The changes in the hind paw thickness in the BEO-treated groups were compared to those in the control group (DMSO) at the same time to evaluate the anti-inflammatory effect of the tested samples. The BEOs or the reference drug (indomethacin) were considered to show an acute anti-inflammatory effect if the extent of reduction in paw edema was statistically significant compared to that in the negative control group (DMSO). The percentage inhibition of inflammation was calculated using the following formulae:

I​ (%)=ΔCc​ −ΔCt​ ΔCc×100​ 

where

I (%): Percentage inhibition at t h.

∆C_C_: Percentage increase in the thickness of the hind paw in the negative control group at t h compared to 0 h.

∆Ct: Percentage increase in the thickness of the hind paw in the positive control group or treated groups at t h compared to 0 h.

### 3.13. Molecular Docking

Molecular docking was used to explore the potential anti-inflammatory targets of *B. lanceolaria* EOs. Twelve main components of the *B. lanceolaria* EO samples from the leaves, stem, and roots (Table 2 and Figure 1) were docked with the key anti-inflammatory protein targets [15,28,29,30,31], including COX-1 (PDB ID: 4O1Z) [45], COX-2 (PDB ID: 6COX) [46], 5-LOX (PDB ID: 6N2W) [47], ALOX15 (PDB ID: [48]), TLR (PDB ID: 2Z7X) [49], NF-κB (PDB ID: 1VKX) [50], TNF-α (PDB ID: 2E7A) [51], iNOS (PDB ID: 3E7G) [52], p38MAPK (PDB ID: 4FA2) [53], JNK (PDB ID: 4Y5H) [54], ERK5 (PDB ID: 4IC7) [55], EGFR (PDB ID: 1M17) [56], ERBB2 (PDB ID: 3PP0) [57], IL-6 (PDB ID: 1P9M) [58], and IL-23R (PDB ID: 3DUH) [59]. All the PDB files were downloaded from the RCSB PDB database “https://www.rcsb.org/ (accessed on 8 May 2022)” and processed using the Discovery Studio Visualizer 2021 [60] and AutoDockTools v1.5.7 [61] to remove water and co-crystalized ligands. Polar hydrogen atoms and Kollman charges were added to the protein structures before converting to the pdbqt file format. The SDF files of the twelve main EO compounds and reference compounds were downloaded from PubChem [62] and converted to pdb format using PyMol 2.4.0 [63]. The structure files were then processed using AutoDock tools v1.5.7 [61] to add Gasteiger charges and define rotatable bonds. The grid box of 20 × 20 × 20 Å (8000 Å^3^) with a 0.375 Å space was generated at the ligand binding site of each protein. For molecular docking, Autodock Vina 1.1.2 [64] with the Lamarckian genetic algorithm was used. The default docking protocol with a rigid protein, flexible ligand, and exhaustiveness value of 8 was used. The lowest binding energy conformation of each ligand was selected for the interaction analysis using PyMol 2.4.0 [63] and the Discovery Studio Visualizer 2021 [60].

### 3.14. Statistical Analysis

The mean values were statistically compared using one-way analysis of variance (ANOVA) with Tukey’s test. The differences were considered significant for *p* < 0.05. The statistical tests were applied using OriginPro, version 8.5.1 (OriginLab Corp, Northampton, MA, USA).

## 4. Conclusions

In this study, the chemical components and their concentrations in the EOs from the leaf, stem, and root samples of *B. lanceolaria,* collected from Vietnam, were successfully elucidated. GC-MS/GC-FID analysis identified 30 compounds in the BEOs, among which, LBEO, SBEO, and RBEO contained 5, 15, and 20 compounds, respectively. Despite the variability among some of the major components in all three types of EOs, all of them showed remarkable anti-inflammatory effects in in vitro and in vivo models of inflammation. LBEO, SBEO, and RBEO inhibited multiple steps in the inflammatory responses of the RAW 264.7 cell model, including (1) NO production, (2) TNF-α, IL-6, iNOS, and COX-2 expression at both mRNA and protein levels, and (3) IκBα and p65 phosphorylation in the NF-κB pathway. In the carrageenan-induced paw edema model, all three EOs inhibited paw edema at both early and delayed phases. These in vivo results are in agreement with the in vitro results, as the inhibition of paw edema at both phases is also associated with the inhibition of key inflammatory components, including COX-1, iNOS production, nitric oxide (NO), and pro-inflammatory cytokines (TNF-α and IL-6). Furthermore, our molecular docking simulation suggested that the chemical components of *B. lanceolaria* EOs target and bind very strongly with many protein components of all three important signaling pathways related to inflammation, including the AA metabolic pathway (COX-1, COX-2, and ALOX15), NF-κB pathway (NF-κB, TNF-α, and iNOS), and MAPK pathway (p38MAPK, JNK, ERK5, EGFR, and ERBB2).

Overall, our results show, for the first time, the detailed chemical composition of BEOs and confirmed their potent anti-inflammatory effects using an in vitro cell model (RAW 264.7), in vivo animal model (carrageenan-induced mouse model of edema) and in silico model (molecular docking of key inflammatory components). These results demonstrate the promising anti-inflammatory potential of BEOs, which can be further utilized to develop effective anti-inflammatory drugs with limited side effects in the future.

## Figures and Tables

**Table 1:** Chemical compositions of the BEOs from the leaves, stem, and roots analyzed using GC-MS/GC-flame ionization detection (FID).

No.	RT (min)	RI	Chemical Composition	Relative Content (%)		
				Leaf (LBEO)	Stem (SBEO)	Root (RBEO)
1	10.22	930	*α*-Thujene	-	-	0.14
2	10.50	939	*α*-Pinene	-	2.28	33.36
3	13.35	1029	*o*-Cymene	38.29	3.35	0.92
4	13.51	1033	Limonene	-	-	0.14
5	15.82	1101	Linalool	-	0.12	-
6	18.23	1169	Albene	-	-	0.72
7	19.22	1198	*α*-Terpineol	-	-	0.14
8	20.38	1231	Nerol	-	-	0.57
9	20.66	1239	Thymol methyl ether	0.24	0.36	21.70
10	20.75	1242	Carvacrol methyl ether	58.28	89.40	-
11	22.50	1293	Thymol	-	-	4.25
12	24.94	1366	*α*-Longipinene	-	-	0.14
13	25.49	1383	Cyclosativene	-	-	0.24
14	25.70	1389	*α*-Copaene	-	0.37	0.65
15	26.47	1413	Petasitene	-	-	0.12
16	27.22	1437	*β*-Caryophyllene	0.26	0.82	0.53
17	27.91	1459	Epi*-β*-Santalene	-	-	0.11
18	27.95	1460	(*Z*)-*β*-Farnesene	-	-	0.19
19	28.30	1471	*α*-Humulene	-	0.17	0.24
20	28.93	1492	Thymol isobutyrate	-	-	29.23
21	29.12	1498	Germacrene D	-	0.16	0.16
22	29.22	1501	*β*-Selinene	-	-	0.68
23	30.28	1537	*δ*-Cadinene	-	0.18	0.26
24	31.54	1579	Geranyl *n*-butanoate	-	-	0.47
25	31.98	1593	Scapanol	-	0.16	0.15
26	32.08	1597	Prenopsan-8-ol	-	-	0.21
27	33.84	1659	*Tau*-Cadinol	-	0.29	0.30
28	34.21	1672	*α*-Cadinol	-	0.27	-
29	38.16	1818	Hexadecanal	-	0.25	-
30	39.76	1881	Hexadecanol	2.05	0.26	-
			**Total**	**99.12**	**98.44**	**95.62**
			Monoterpenes	96.81	95.51	61.94
			Sesquiterpenes	0.26	2.13	4.45
			Other	2.05	0.51	29.23

RT = retention time; RI = retention index.

**Table 2:** Docking energy of the 12 main compounds with target proteins.

Compounds		Calculated Affinity Energy (kcal/mol)														
No.	Name	COX-1	COX-2	5-LOX	ALOX15	TLR	NF- κB p50p65	TNF-α	iNOS	p38MAPK	JNK	ERK5	EGFR	ERBB2	IL-6	IL-23R
**2**	α-Pinene	−6.1	−6.2	−4.9	−6.4	−3.5	−4.9	−4.9	−5.3	−5.9	−5.5	−5.3	−5.5	−6.4	−4.7	−4.3
**3**	o-Cymene	−6.4	−6.3	−5.0	−6.2	−4.0	−5.9	−4.7	−5.8	−6.3	−5.7	−5.7	−5.6	−6.3	−4.7	−4.3
**9**	Thymol methyl ether	−6.4	−6.7	−5.5	−6.4	−4.0	−5.9	−5.4	−5.5	−6.1	−5.8	−5.8	−5.6	−6.6	−5.0	−4.5
**10**	Carvacrol methyl ether	−6.2	−6.9	−5.8	−6.7	−4.1	−6.2	−5.3	−6.2	−6.5	−5.8	−6.0	−6.0	−6.9	−4.9	−4.4
**11**	Thymol	−6.3	−6.5	−5.7	−6.3	−4.0	−6.1	−5.3	−6.2	−6.6	−5.6	−6.0	−5.9	−6.9	−4.9	−4.4
**14**	*α*-Copaene	**−7.5**	**−7.7**	−6.4	**−7.6**	−4.4	−5.8	−6.3	−6.1	**−7.4**	**−7.2**	**−7.3**	−6.9	**−7.2**	−5.4	−5.5
**16**	*β*- Caryophyllene	**−9.0**	**−8.1**	−6.2	**−8.2**	−5.2	−6.7	−6.6	**−7.1**	**−8.2**	**−8.0**	**−7.2**	**−7.4**	**−7.7**	−6.1	−5.7
**19**	*α*-Humulene	**−7.2**	**−7.7**	−6.1	**−7.4**	−5.0	−5.8	−6.7	−6.1	**−7.8**	**−7.1**	−6.9	**−7.2**	**−7.3**	−5.6	−5.7
**20**	Thymol isobutyrate	**−7.6**	**−7.7**	−6.1	**−7.6**	−4.7	**−7.0**	−6.1	−6.4	**−7.1**	−6.5	−6.7	−6.5	**−8.0**	−5.3	−5.2
**23**	*δ*-Cadinene	**−7.4**	**−7.8**	−6.1	**−7.7**	−4.7	**−7.6**	−6.5	−6.5	**−8.5**	**−7.4**	**−7.5**	**−7.0**	**−8.3**	−5.9	−5.8
**27**	*Tau*-Cadinol	**−7.0**	**−7.8**	−6.4	**−9.1**	−5.0	**−7.1**	−6.6	−6.3	**−7.4**	**−7.3**	**−7.0**	**−7.5**	**−8.2**	−6.2	−6.1
**30**	1-Hexadecanol	−5.9	−5.7	−4.4	−4.7	−3.1	−4.6	−4.6	−5.5	−5.7	−5.0	−4.8	−4.7	−6.4	−4.1	−3.6
**Reference ligands**	**Docking scores**	−9.4	−11.0	−7.1	−7.3	NA	NA	NA	−8.3	−7.8	−7.8	−8.0	−7.4	−11.4	NA	NA
	**Name**	Meloxi-cam	Celeco-xib	30Z	RS7	NA	NA	NA	AT2	SB0	PDB ID:519	ANP	AQ4	03Q	NA	NA

**Table 3:** Primers for qRT-PCR.

No.	Name	Sequence (5′-3′)	Length (nm)	Size (bp)	Reference
1	β-actin-F	ATCACTATTGGCAACGAGCG	20	191	Lee, 2016 [41]
2	β-actin-R	TCAGCAATGCCTGGGTACAT	20		
3	iNOS-F	TTCCGAAGTTTCTGGCAGCAGC	25	491	Li, 2011 [42]
4	iNOS-R	TGTCAGAGCCTCGTGGCTTTGG	25		
5	COX2-F	GGAGTCTGGAACATTGTGAAC	21	156	Tian, 2021 [43]
6	COX2-R	GTAGTAGGAGAGGTTGGAGAAG	22		
7	TNF-α-F	ATGAGCACAGAAAGCATGATC	21	276	Kim, 2016 [9]
8	TNF-α-R	TACAGGCTTGTCACTCGAATT	21		
9	IL-6-F	GAGGATACCACTCCCAACAGACC	23	141	Lee, 2016 [41]
10	IL-6-R	AAGTGCATCATCGTTGTTCATACA	24		

## Data Availability

Data are available upon reasonable request.

## Supplementary Materials

The following supporting information can be downloaded at: https://www.mdpi.com/article/10.3390/molecules27227839/s1, Figure S1. GC-MS chromatogram of the essential oils of B. lanceolaria leaf (LBEO); Figure S2. GC-MS chromatogram of the essential oils of B. lanceolaria stem (SBEO); Figure S3. GC-MS chromatogram of the essential oils of B. lanceolaria root (RBEO).

## Sample Availability

Samples are available from the authors.
