# Supplementary material for: Essential Oils from the Leaves, Stem, and Roots of Blumea lanceolaria (Roxb.) Druce in Vietnam: Determination of Chemical Composition, and In Vitro, In Vivo, and In Silico Studies on Anti-Inflammatory Activity
Source: Molecules. 2022 Nov 14;27(22):7839. doi: 10.3390/molecules27227839 (PMC9697122; doi:10.3390/molecules27227839)
Supplement: Supplementary file 1 [file molecules-27-07839-s001.zip › molecules-1987335-supplementary.pdf]

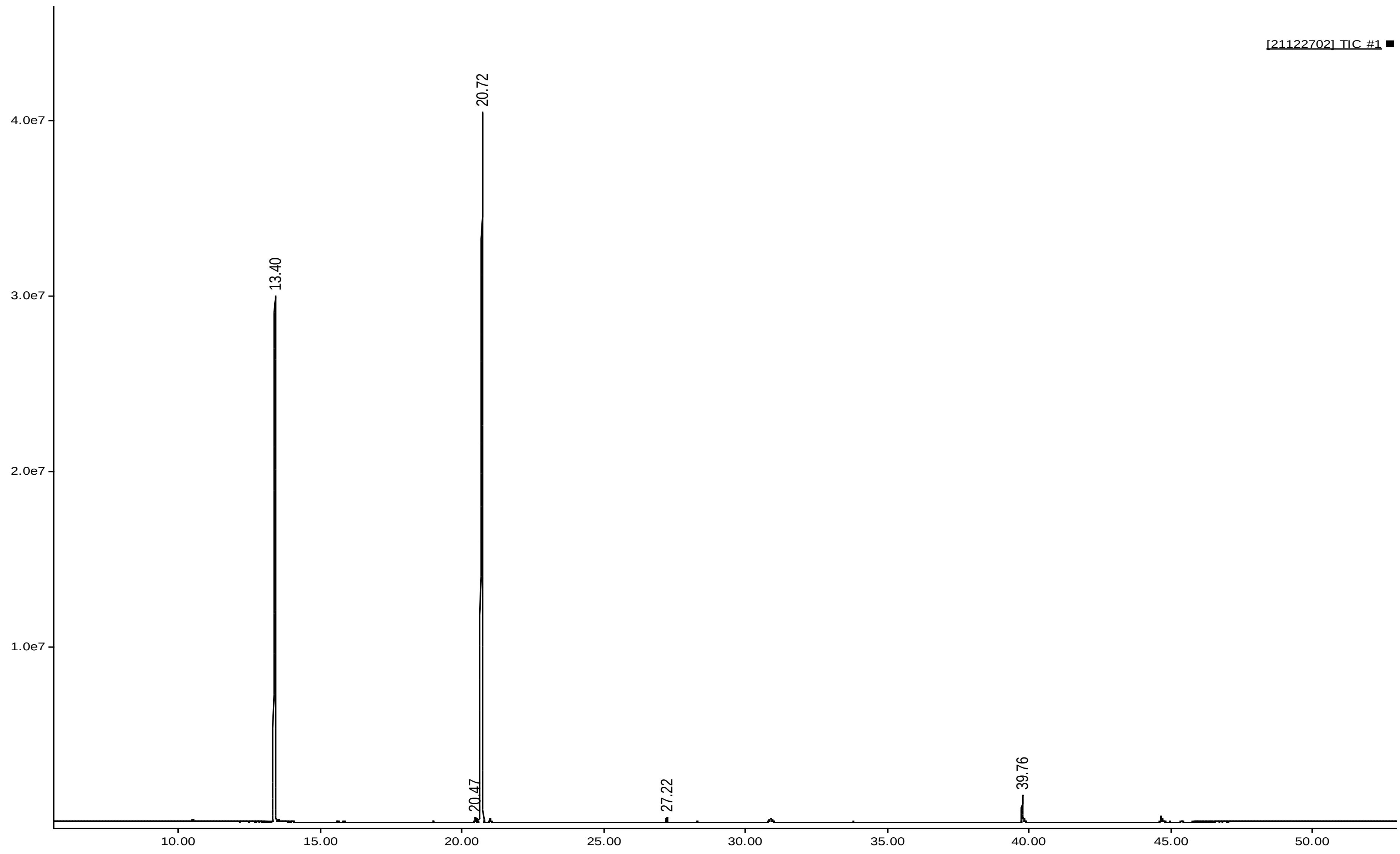

Figure S1. GC-MS chromatogram of the essential oils of *B. lanceolaria* leaf (LBEO)

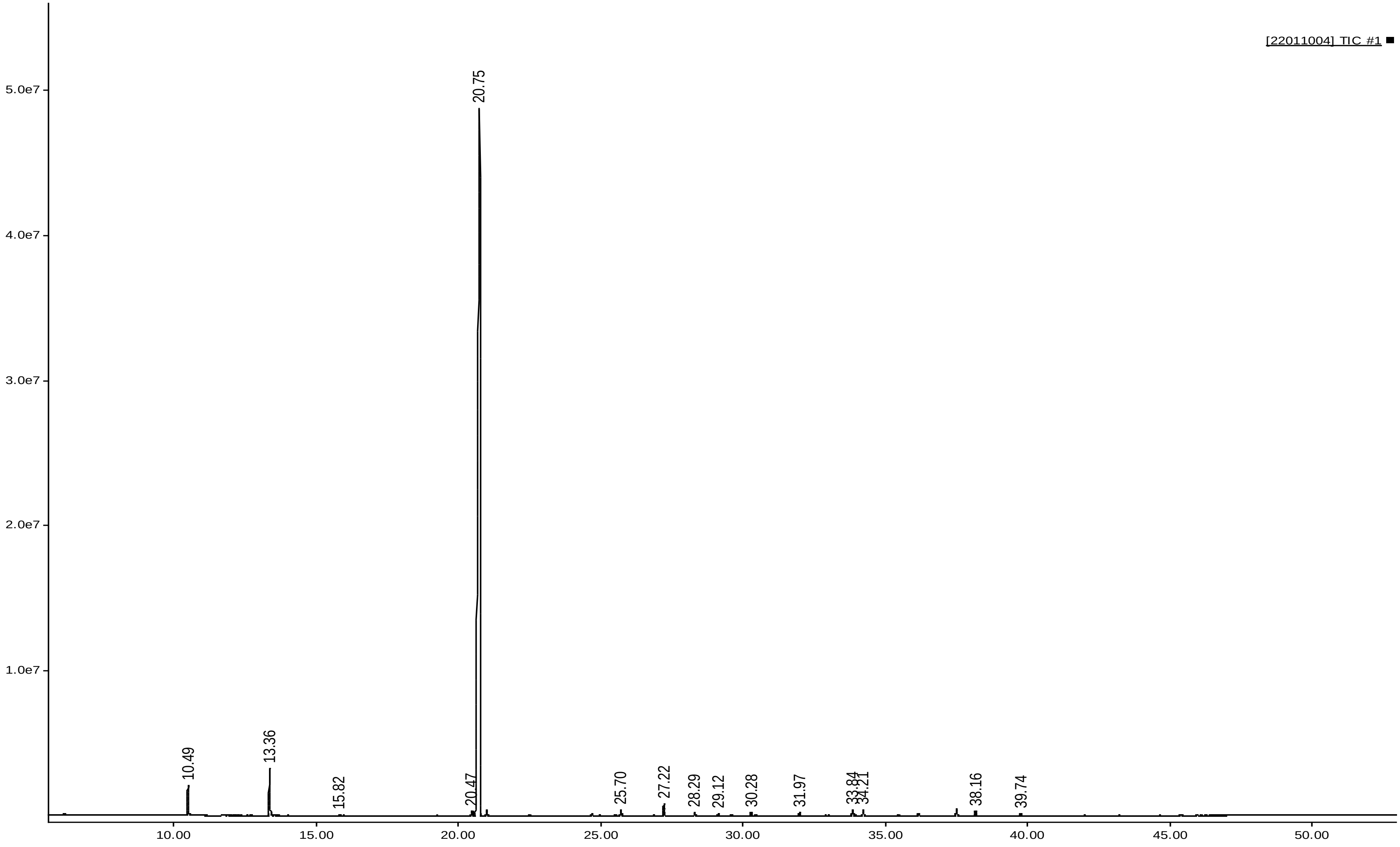

Figure S2. GC-MS chromatogram of the essential oils of *B. lanceolaria* stem (SBEO)

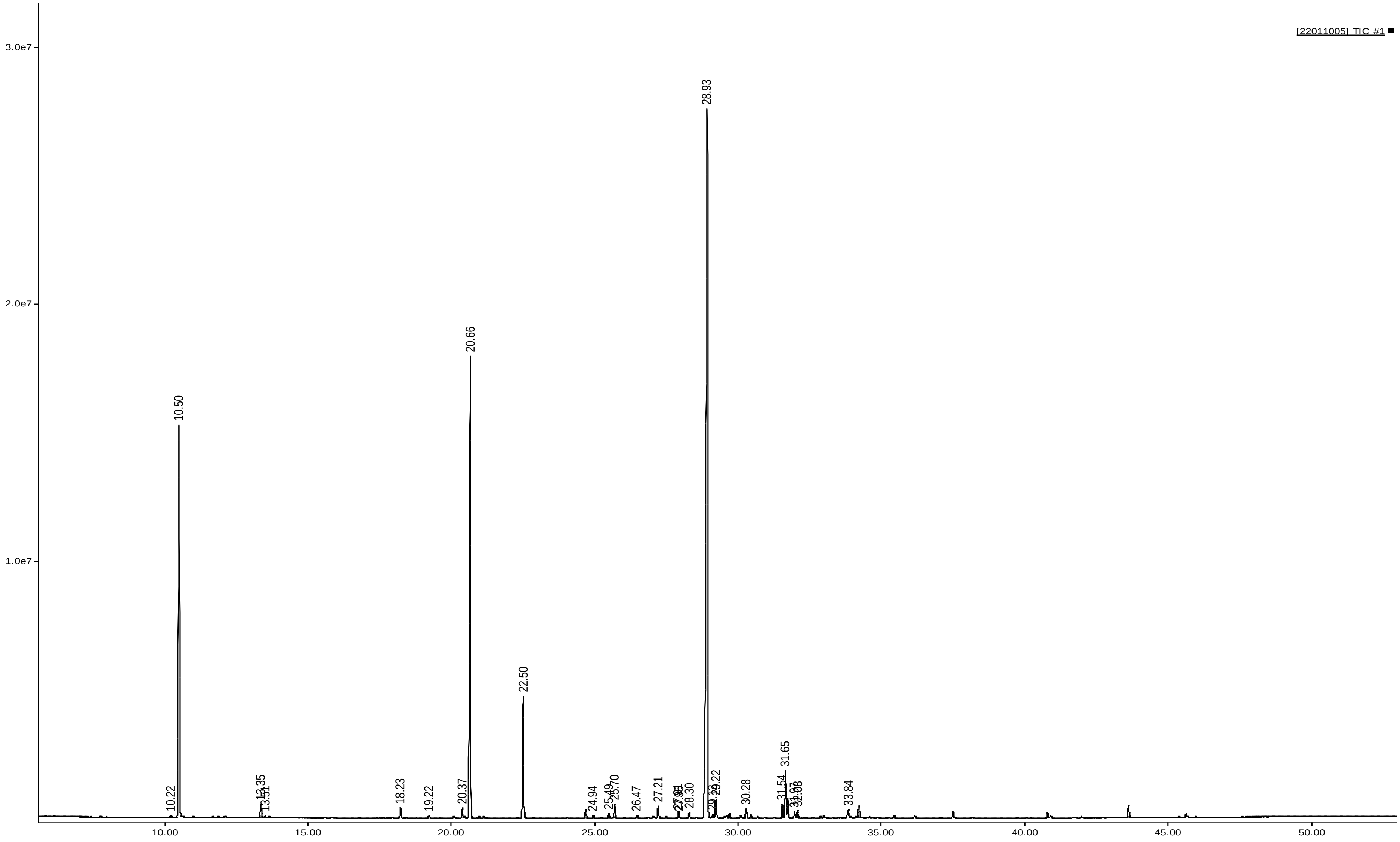

Figure S3. GC-MS chromatogram of the essential oils of *B. lanceolaria* root (RBEO)
